# ULTRASONOGRAPHY OF THE CARTILAGINOUS PATELLA IN PEDIATRIC PATIENTS: A CASE SERIES

**DOI:** 10.1590/1413-785220253305e291176

**Published:** 2025-09-22

**Authors:** Leila Pereira Tenório, José Batista Volpon, Marcello Henrique Nogueira-Barbosa

**Affiliations:** 1 Universidade de São Paulo (FMRPUSP), Faculdade de Medicina de Ribeirão Preto, Hospital das Clinicas, Ribeirao Preto, São Paulo, SP, Brazil.; 2 Universidade de São Paulo (FMRPUSP), Faculdade de Medicina, Departamento de Ortopedia e Anestesiologia, Ribeirão Preto, São Paulo, SP, Brazil.; 3 Universidade de São Paulo (FMRPUSP), Faculdade de Medicina, Departamento de Imagens Médicas, Hematologia e Oncologia Clinica, São Paulo, SP, Brazil.

**Keywords:** Patella, Patellar Dislocation, Nail-Patella Syndrome, Ultrasonography, Congenital Abnormalities, Patela, Luxação Patelar, Síndrome da Unha-Patela, Ultrassonografia, Anormalidades Congênitas

## Abstract

**Objective:**

To analyze cases with clinical suspicion of patellar abnormalities, before ossification of the patella and to characterize the spectrum of abnormalities of the cartilaginous infantile patella by ultrasonography.

**Methods:**

Retrospective study using the keyword "patella" in ultrasonography reports in the Radiology Information System (RIS). The main researcher performed patellar measurements in the group of patients and in a control group (9 patients) without clinical or ultrasonography abnormalities.

**Results:**

Twelve patients with suspected patellar abnormalities were identified, with a mean age of 9 months and 4 days (±1.9 years), 75% male.

**Findings:**

dislocation or subluxation associated with patellar hypoplasia (7 knees), low lying patella and patellar hypoplasia (2), unilateral patellar agenesis (1), bilateral patellar agenesis (1), patellar instability in dynamic assessment and absence of patellar morphological changes (1). In two patients, ultrasonography was negative. The craniocaudal diameter of the hypoplastic patellas measured 0.94 cm ± 0.24 cm and in the control group 1.24 cm and ±0.12 cm (p<0.01). The Insall-Salvati index adapted for ultrasonography measured 0.63±0.07 for the low lying patella and 0.93±0.16 in the control group (p=0.004).

**Conclusions:**

Ultrasonography was useful to characterize abnormalities of the cartilaginous patella, and the most frequent findings were instability and hypoplasia. ***Level of Evidence IV; Case Series.***

## RESUMO

**Objetivo:**

Analisar casos com suspeita clínica de alterações patelares, antes da ossificação da patela ossificação da patela e caracterizar o espectro de anormalidades da patela infantil cartilaginosa pela ultrassonografia.

**Métodos:**

Estudo retrospectivo a partir da palavra-chave "patela" nos relatórios de ultrassonografia no Sistema de Informação em Radiologia (RIS). A pesquisadora principal realizou medidas patelares no grupo de pacientes e em grupo controle de 9 pacientes sem anormalidades clínicas ou ultrassonográficas.

**Resultados:**

Foram identificados doze pacientes com suspeita de alterações patelares, com idade média de 9 meses e 4 dias (±1,9 anos), 75% do sexo masculino.

**Achados:**

luxação ou subluxação associada a hipoplasia patelar (7 joelhos), patela baixa e hipoplasia patelar (2), agenesia patelar unilateral (1), agenesia patelar bilateral (1), instabilidade patelar na avaliação dinâmica e ausência de alterações morfológicas patelares (1). Em dois pacientes a ultrassonografia não mostrou alterações. O diâmetro crânio caudal das patelas hipoplásicas mediu 0,94 cm ± 0,24 cm e no grupo controle 1,24 cm e ±0,12 cm (p<0,01). O índice Insall-Salvati adaptado para ultrassonografia das patelas baixas mediu 0,63±0,07 e no grupo controle 0,93±0,16 (p=0.004).

**Conclusões:**

A ultrassonografia foi útil para caracterizar alterações da patela cartilaginosa e os achados mais frequentes foram instabilidade e hipoplasia da patela. ***Nível de Evidência IV, Série de Casos.***

## INTRODUCTION

The patella is the largest sesamoid bone in the skeleton, connecting at the proximal pole with the quadriceps femoris tendon and distally with the patellar ligament. It helps stabilize the knee, increases knee extension strength, improves the mechanical performance of the quadriceps, and protects the articular cartilage that lines the joint.^1^ In humans, the patella undergoes endochondral ossification, which ends in late adolescence.^2^

The patella is cartilaginous in individuals under two years of age and is therefore not easily identifiable on X-rays. For this reason, the most appropriate imaging methods for its investigation are ultrasonography or magnetic resonance imaging.^2^ The advantage of ultrasonography over magnetic resonance imaging is the possibility of performing dynamic maneuvers, studying different degrees of flexion or extension, faster execution, no need for patient sedation, and, additionally, lower cost.^3^

The absence or hypoplasia of the patella is a rare congenital anomaly that can occur in isolation, as part of specific syndromes, or associated with disorders such as trisomy 8.^4^ The prevalence of patella agenesis or hypoplasia in newborns is difficult to determine because this structure is completely cartilaginous at birth,^5^ but these conditions mainly result from developmental defects. There are no objective criteria for radiographic diagnosis of patella hypoplasia or reference measurements for different age groups, which means that assessment is subjective.^2^

Clinical diagnosis in young children is difficult because the absent, dislocated, hypoplastic, or unstable patella is difficult to palpate and evaluate. However, early diagnosis in the neonatal phase is important because it enables the investigation of genetic syndromes,^2^ and allows for early planning and treatment.^3^

The aim of this study was to analyze cases with clinical suspicion of patellar abnormalities in children who had not yet undergone patellar ossification and to identify ultrasound abnormalities.

## MATERIALS AND METHODS

Study approved by the Institutional Research Ethics Committee, with waiver of informed consent (CAAE: 19385819.3.0000.5440).

### Patient selection

A retrospective search was conducted for cases with clinical suspicion of patellar abnormalities based on knee ultrasound reports identified in the institution's Radiology Information System (RIS). The search term was "patella". 750 reports containing this keyword were found, including the words "patellar" and "suprapatellar" which contain the word "patella." Examinations of adult patients were excluded. Next, the reports and images of pediatric patients were reviewed and correlated with the clinical history in the electronic medical record. Ultimately, only patients with clinical suspicion of patellar changes (n = 12) were included. The exclusion criteria were: previous patella surgery (in one case) and a lack of images in the digital archiving system (PACS) in two cases.

### Ultrasonography examination protocol

**Figure 1:**
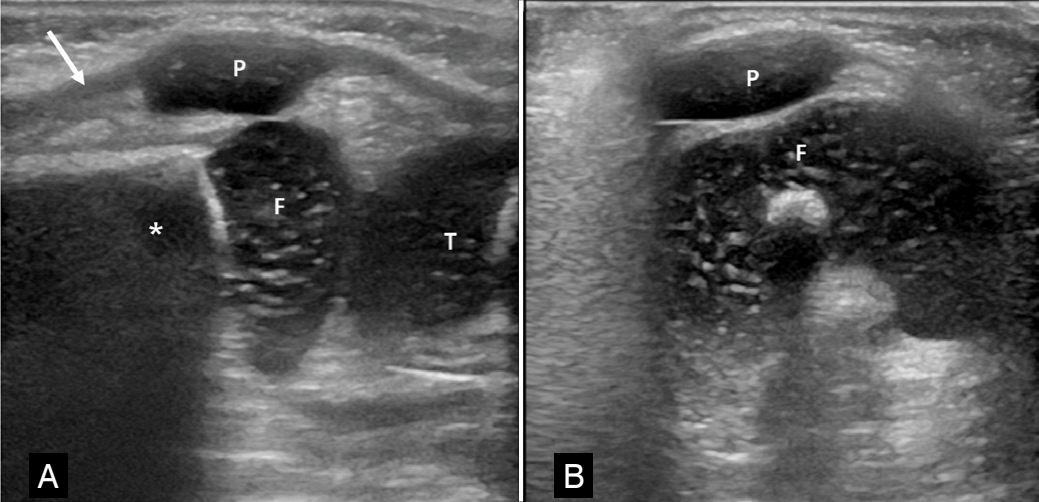
Normal examination to demonstrate the protocol in a male patient aged 2 months. The longitudinal section (A) shows the quadriceps tendon (arrow), the cartilaginous patella (P), the distal metaphysis of the femur (*), the distal epiphysis of the femur (F), and the proximal epiphysis of the tibia (T). The image in the axial plane (B) shows congruent patella (P) and femoral trochlea (F).

All examinations were performed according to the following protocol. Patients were placed in the supine position and images of the patella were acquired in longitudinal and axial slices (transverse to the long axis of the patella), with the knee in flexion and extension. Cases with stiff knees were examined in the position in which they were found, without forcing correction of the deformity. Initially, the extensor mechanism was evaluated in longitudinal sections, where the quadriceps tendon, the patella, and the entire length and insertion of the patellar ligament were located (Figure 1). The patella and femoral trochlea were documented in the axial sections. If the patella was not identified in the anterior region of the knee, the lateral aspect of the knee was scanned, looking for possible images of the dislocated cartilaginous patella and in continuity with the extensor mechanism. Subsequently, if the patella was still not found, the medial aspect of the knee was assessed to confirm that the patella was not present.

### Measurement of the patellae

The musculoskeletal radiology fellow measured the largest diameter (cephalocaudal) of the cartilaginous patellae previously classified as hypoplastic based on the senior radiologist's subjective assessment, using images available in the institution's digital image archiving and distribution system (PACS). He also performed retrospective measurements of the diameters of the cartilaginous patella and patellar tendon in cases subjectively classified as "low patella" to measure the Insall-Salvati index in a similar manner to that used for measuring the index on radiographs.

For comparison, images from a control group were used, retrospectively identified in the institutional PACS, belonging to nine patients (18 patellas) with no clinical or ultrasonographic abnormalities in the knees, and whose examinations had not been indicated for knee evaluation.

### Evaluation of medical records and images

After collecting data from medical records (Table 1), ultrasound images were reviewed by a musculoskeletal radiology fellow and a musculoskeletal radiologist with 22 years of experience. The criterion for patella dislocation in ultrasound images was based on complete loss of contact between the articular surfaces of the patella and femoral trochlea, and subluxation was considered when there was partial loss of contact between the articular surfaces.

**Table 1:** Characterization of patients evaluated by ultrasound and with clinical suspicion of patellar abnormality.

Age and gender	Physical findings	Main clinical suspicion	Ultrasound findings
2 months 8 days Male	Congenital hip dislocation and bilateral cryptorchidism	Nail-patella syndrome	Hypoplasia and lateral dislocation of the right patella
6 days Women	Bone dysplasia, hip and knee dislocation	Arthrogryposis Larsen syndrome	Patellas without morphological alterations, but unstable during dynamic maneuvers.
12 days Women	Breech presentation, congenital hip dysplasia, congenital clubfoot	Congenital malformation	Hypoplasia and low lying patellas
4 months and 15 days Male	Total hemimelia of the tibia and dislocation of the right knee	Congenital malformation	Right patella agenesis and distal femur dysplasia
2 months and 14 days Male	Myelomeningocele and congenital clubfoot	Chiari type II and hydrocephalus	Patellae with no echographic changes
6 years and 9 months Male	Proximal femur deficiency and right fibular hemimelia	Congenital malformation	Right lateralized patella with trochlear dysplasia
27 days Male	Left genu recurvatum, contracture in hip adduction and elbow flexion, clinodactyly	VACTERL	Hypoplasia and lateral dislocation of the left patella
3 months and 10 days Male	Meromelia, deformity in flexion of the hips and knees. Absence of both arms and forearms	Arthrogryposis Fuhrmann syndrome	Patella agenesis
2 months and 23 days Male	Imperforate anus, acetabular dysplasia, and cryptorchidism	Intrauterine disruptive syndrome	Hypoplastic patellas and lateral subluxation on the right
1 year Male	Proximal focal deficiency of the femur with fibular hemimelia on the left	Femur-fibula-ulna syndrome Fuhrmann syndrome	Hypoplastic left patella and lateral dislocation associated with femoral trochlear dysplasia
25 days Male	Partial tibial hemimelia and focal proximal deficiency of the right femur, left hip dislocation	Malformations secondary to uncontrolled maternal diabetes	Hypoplastic right patella and medial dislocation associated with femoral trochlear dysplasia
1 month and 3 days Women	Contraction of the hips and genu recurvatum	Larsen syndrome	Anterior dislocation of the tibiae, hypoplastic patellae, and low position

## RESULTS

**Figure 2:**
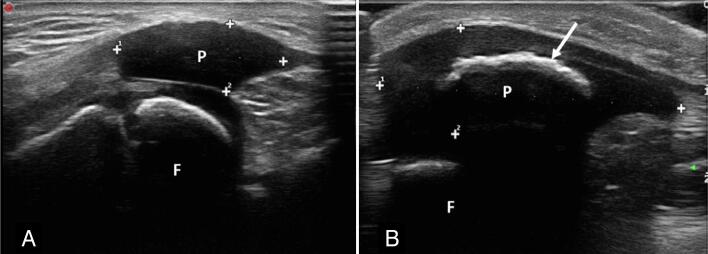
Male patient, aged 6 years and 9 months, with a previous diagnosis of right fibular hemimelia, complete agenesis of the fibula, and shortening of the ipsilateral femur and tibia. The right patella (A) was not easily identifiable on physical examination. The longitudinal ultrasound image shows patellar hypoplasia and absence of ossification, with the patella still cartilaginous, while the left patella (B) already shows ossification in the central region (arrow).

The average age of patients in the study group at the time the tests were performed was 9 months and 4 days, with a standard deviation of 1.9 years. Most patients were male (75%). In nine cases, ultrasound was performed before four months of age (75%), with four cases evaluated during the neonatal period (33%). In three patients, the examination was requested after this age due to significant deformities of the lower limbs; two of them had limb dysmetria, proximal femur shortening associated with fibular hemimelia and total hemimelia of the right tibia, conditions diagnosed in previous radiographs. (Figure 2)

In all cases with patellar abnormalities, there were also other musculoskeletal system changes, such as retroverted knees, hip dysplasia, congenital clubfoot, and bilateral meromelia, with the hands implanted directly into the chest.

**Figure 3:**
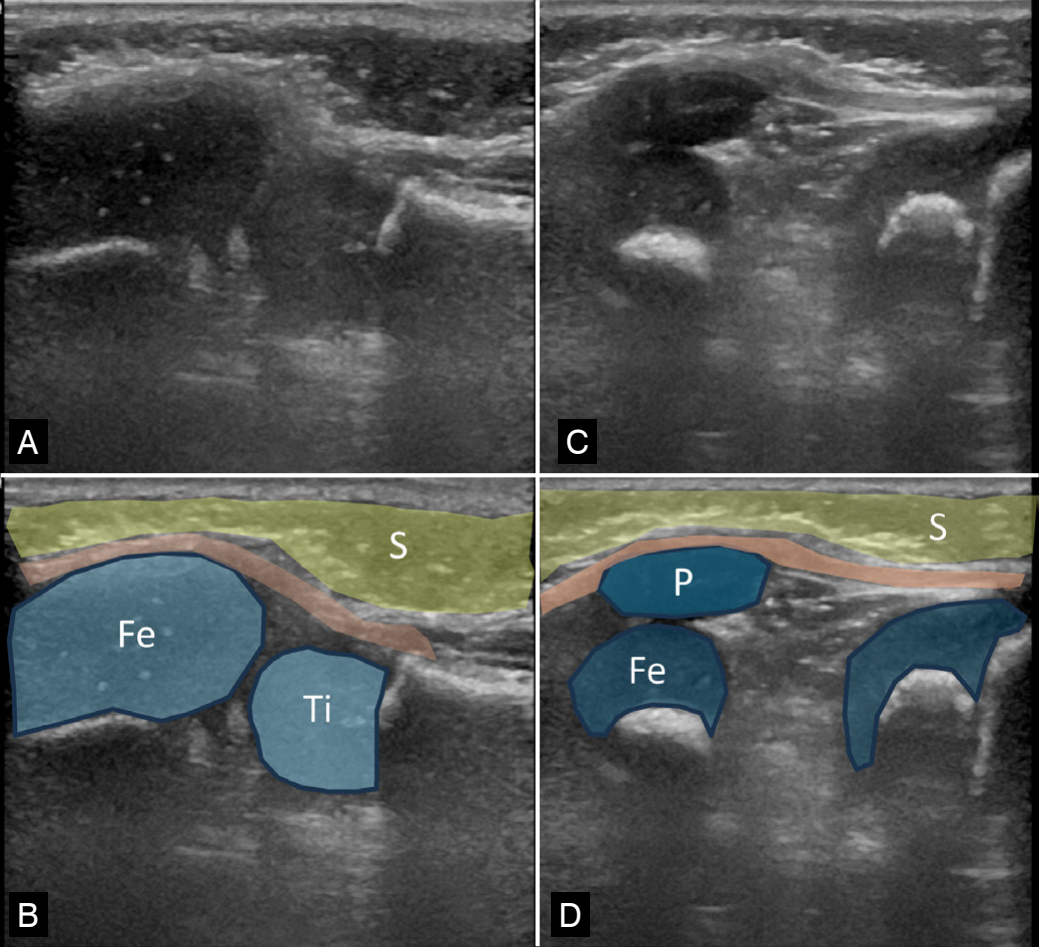
Male patient, four months old, diagnosed with right fibular hemimelia and knee dislocation. The patella was not palpable, and ultrasound confirmed the diagnosis of agenesis of the right patella (A). On the left side (C), the patella is present, and the longitudinal section shows the patella. In B and D, the subcutaneous tissue is marked in yellow, the epiphyseal cartilages in blue, and the extensor mechanism in orange; Fe = femur, P = patella, S = subcutaneous tissue, Ti = tibia.

Of the twelve patients included (Table 1), two had normal patellas, one had congenital clubfoot, and the other was diagnosed with bone dysplasia and dislocation of the hips and knees. Unilateral patella agenesis was found in one case (16% of patients) (Figure 3). Patella dislocation was found in five cases (41% of the sample), four of which were associated with patellar hypoplasia, and only one patient did not show a size reduction.

Of the dislocation cases, four presented lateral patella dislocation and only one presented medial dislocation. Two cases of congenitally low lying patellas associated with hypoplasia, but without dislocation, were also found.

Most patients had clinical suspicion of genetic syndromes or congenital malformations, including arthrogryposis, nail-patella syndrome, and VACTERL association or syndrome (V = vertebral anomalies, A = anal atresia, C = cardiovascular anomalies, T = tracheoesophageal fistula, E = esophageal atresia, R = renal and/or radial anomalies, L = limb defects). A complete description of the associated abnormalities in each patient is provided in Table 1. The Shapiro-Wilk test revealed that patellar diameter measurements did not follow a normal distribution, whereas ultrasonographic Insall-Salvati index measurements exhibited a normal distribution. The cranial-caudal diameter of the "hypoplastic patellae" measured 0.94 cm ± 0.24 cm, and in the control group it measured 1.24 cm ± 0.12 cm (Mann-Whitney p < 0.01). The Insall-Salvati index, adapted for ultrasound of "low lying patellas," measured 0.63 ± 0.07 in the study group and 0.93 ± 0.16 in the control group (p = 0.004).

## DISCUSSION

This series of cases illustrates the spectrum of cartilaginous patella abnormalities identified by ultrasonography in the pediatric population. The most frequently detected abnormality was lateral patellar instability, which presented as dislocation, subluxation, or dynamic instability. In most cases of patellar instability, this anomaly was accompanied by hypoplasia. However, we found patellar hypoplasia without signs of instability and vice versa (one case each). Ultrasonography was useful for identifying patellar agenesis and ruling out other patellar abnormalities, such as hypoplasia or dislocation.

There is scarce literature on the role of ultrasonography in the evaluation of the immature patella. Miller et al. described a series of three children with patellar abnormalities.^5^ Another series of four children (two neonates, one three-year-old, and one four-year-old) reinforced the potential of ultrasonography for studying congenital displacement of the cartilaginous patella in the pediatric population.^6^ Congenital malformations of the patellae may manifest as complete agenesis, hypoplasia, or dislocation. The evaluation of patellar changes in children under two years of age should be performed using ultrasound due to the lack of ossification in this age group.^2^ Patellar malformations may manifest shortly after birth as external rotation of the tibia, valgus knee, and flexion contracture. However, in some cases, diagnosis may be delayed until early childhood. In less severe cases, where function is less impaired, diagnosis may be delayed, sometimes not occurring until adolescence or adulthood. The most common complaint among these patients in adulthood is pain due to instability and progression to osteoarthritis.^7^ Congenital malformations of the lower limbs without associated upper limb anomalies occur in 1 in every 10,000 live births.^8^ More than thirty-five dysmorphic entities are associated with agenesis or reduction of the patella according to the Winter-Baraitser dysmorphology database (WBDD), version 1.0.4, London Medical Databases.^1^ Among the main malformation syndromes associated with patellar agenesis are nail-patella syndrome and small patella syndrome. According to Vanlerberghe et al.,^2^ for these two syndromes, hypoplasia or agenesis of the patella is a constant or highly frequent feature that constitutes a primary clinical indicator aiding in diagnosis. On the other hand, patellar dislocation is associated with Rubinstein-Taybi and William-Beuren syndromes.^9^ For other rare diseases, other clinical features are more important, but the identification of patella anomalies may be indicative of clinical genetic diagnosis.^2^

We found no data in the literature on the prevalence of isolated cases of patellar abnormalities or those associated with genetic syndromes. In the present study, all patients with patellar malformations had other clinical conditions, and none had isolated patellar malformation.

Cormier-Daire, V. et al reported seven cases (six boys and one girl) presenting with congenital absence of the patella, genital and renal anomalies, dysmorphic features, and mental retardation, and suggested classifying them as a new clinical entity named genitopatellar syndrome. In this study, patellar agenesis was confirmed in two patients over the age of six years by radiography.^4^

Miller et al. reported three cases of ultrasonographic evaluation in patients with congenital anomalies of the extensor mechanism, one of whom presented unilateral patellar subluxation and hypoplasia, the second with normal patellar development with patellar tendon hypotrophy and superior patellar dislocation, and the third with bilateral patellar agenesis with a clinical diagnosis of nail-patella syndrome.^5^ We did not find objective or quantitative criteria in the literature for defining patellar hypoplasia. In the current study, the diagnostic impression of hypoplasia was based on the interpretation of a senior radiologist with over 20 years of experience, as well as comparative analysis with the contralateral side when possible. We found a statistically significant difference in the cranial-caudal diameter of the patellae measured by ultrasound between patellae qualitatively classified as hypoplastic and control knees.

Studies describing patients with congenitally low lying patella do not report objective or quantitative criteria for obtaining this diagnosis, and presumably rely on the observer's experience and comparison with the contralateral side when possible.

Of the twelve patients in our study, two had bilateral congenitally low lying patellae (patella baja) based on qualitative assessment. We found a statistically significant difference in the Insall-Salvati index adapted for ultrasonography between patellas considered qualitatively as low lying patellas and patellas from a control group of knees.

According to Dejour et al., low lying patella is usually a complication of trauma, previous knee surgery, or neuromuscular disease, and is rarely a congenital condition.^10^ There is a case report of two siblings with Larsen syndrome who had morphological abnormalities of the knees, including low patellae.^11^ Another study evaluated 34 patients with congenital multiple arthrogryposis and found that four of them had low patellas; however, the authors did not report the criteria used for this classification.^12^ In our case series, both patients with low lying patellas had *genu recurvatum*.

In recent years, several human genes important for patella development have been discovered through the study of malformation syndromes. These recent data show that patellar anomalies may result from the dysregulation of various cellular or developmental processes. Investigation of the ultrasonographic phenotype and genetics of patellar anomalies could potentially enhance our understanding of lower limb development and reveal information about the genes responsible for the normal development of other organic systems. Ultrasound is the recommended examination for evaluating the immature skeleton in cases of suspected hip dysplasia,^13–17^. It can be used to evaluate fractures of the ossification centers of the elbow^18^ and the proximal ossification centers of the humerus.^19^ Ultrasound should be considered for investigating cartilaginous structures in the pediatric age group due to its low cost and the possibility of dynamic evaluation without the need for ionizing radiation or sedation.^5^ MRI images can also be used to study the cartilaginous anatomical structures of the immature skeleton, but with relative disadvantages due to lower availability, the need for sedation, generally static evaluation, and the fact that comparison with the contralateral side is often not possible.^2^

The evaluation of patellar cartilage by ultrasound enables the identification or exclusion of patellar abnormalities that may have a potential impact on treatment choice and prognosis for patients. Patella agenesis or hypoplasia can cause pain, difficulty walking, running, or climbing stairs. Patellofemoral instability can progress to recurrent dislocations, accompanied by painful episodes and early osteoarthritis. Early diagnosis can prevent late sequelae and reduce the costs associated with medical care and the need for late correction of lower limb deformities.

The main limitations of this study are its retrospective design and small sample size. However, our study can contribute to the literature, given the scarcity of studies and the small sample sizes of those available.^6^ In addition, most of our cases were not analyzed by molecular genetics to advance the etiology of genetic diseases, due to the difficulty of obtaining these tests.

Despite these limiting factors, the findings presented in this study confirm that ultrasonography can be a useful method for the early evaluation of children with suspected congenital patellar abnormalities. This is particularly relevant in the first years of life, when the patella has not yet ossified, making radiographic evaluation difficult. We did not find quantitative or objective criteria in the literature to establish the diagnosis of low lying patella and patellar hypoplasia. This limitation, as found in the literature, reinforces the need for prospective studies with a larger number of children evaluated by ultrasonography to establish criteria for normal pediatric patellar cartilage.

## CONCLUSION

Ultrasonography was useful for characterizing abnormalities in the cartilaginous patella in the pediatric population. The most frequent anomaly was lateral patellar instability, identified by dislocation, subluxation, or dynamic instability. Ultrasonography also allows differentiation between patellar agenesis and dislocation associated with patellar hypoplasia.
